# Severe Monkeypox in Hospitalized Patients — United States, August 10–October 10, 2022

**DOI:** 10.15585/mmwr.mm7144e1

**Published:** 2022-11-04

**Authors:** Maureen J. Miller, Shama Cash-Goldwasser, Grace E. Marx, Caroline A. Schrodt, Anne Kimball, Kia Padgett, Rebecca S. Noe, David W. McCormick, Joshua M. Wong, Sarah M. Labuda, Brian F. Borah, Isaac Zulu, Amimah Asif, Gurpreet Kaur, Janet M. McNicholl, Athena Kourtis, Andrew Tadros, Sarah Reagan-Steiner, Jana M. Ritter, Yon Yu, Patricia Yu, Rachel Clinton, Corrine Parker, Eleanor S. Click, Johanna S. Salzer, Andrea M. McCollum, Brett Petersen, Faisal S. Minhaj, Ericka Brown, Michael P. Fischer, Robert L. Atmar, Andrew R. DiNardo, Ya Xu, Cameron Brown, Jerry Clay Goodman, Ashley Holloman, Julia Gallardo, Hanna Siatecka, Georgia Huffman, John Powell, Philip Alapat, Pralay Sarkar, Nicola A. Hanania, Or Bruck, Steven D. Brass, Aneesh Mehta, Alexandra W. Dretler, Amanda Feldpausch, Jessica Pavlick, Hillary Spencer, Isaac Ghinai, Stephanie R. Black, Laura N. Hernandez-Guarin, Sarah Y. Won, Shivanjali Shankaran, Andrew T. Simms, Jemma Alarcón, Jesse G. O’Shea, John T. Brooks, Jennifer McQuiston, Margaret A. Honein, Siobhán M. O’Connor, Kevin Chatham-Stephens, Kevin O’Laughlin, Agam K. Rao, Elliot Raizes, Jeremy A. W. Gold, Sapna Bamrah Morris, CDC Severe Monkeypox Investigations Team, CDC Severe Monkeypox Investigations Team, Shelby Duessel, Darren Danaie, Angela Hickman, Brynn Griffith, Haddijatou Sanneh, Helena Hutchins, Christine Phyathep, Ann Carpenter, Victoria Shelus, Julia Petras, Ian Hennessee, Meryl Davis, Cristin McArdle, Patrick Dawson, Bruce Gutelius, Kris Bisgard, Karen Wong, Romeo R. Galang, Kiran M. Perkins, Thomas D. Filardo, Whitni Davidson, Christy Hutson, David Lowe, Jason E. Zucker, David A. Wheeler, Lucy He, Aabha K. Jain, Oleksandr Semeniuk, Dev Chatterji, Marnie McClure, Lucy X. Li, Jona Mata, Sasha Beselman, Sara L. Cross, Barbara Menzies, Marina Keller, New York, Vishnu Chaturvedi, New York, Andrea Thet, Ryan Carroll, Courtney Hebert, Gopi Patel, Vani Gandhi, Alexandra Abrams-Downey, Mehmood Nawab, Emily Landon, Gregory Lee, Emma Kaplan-Lewis, Cyndee Miranda, Anna E. Carmack, Edward C. Traver, Susana Lazarte, Trish M. Perl, Jeremy Chow, Ellen Kitchell, Ank Nijhawan, Onaizah Habib, Allen Bernus, Gabriela Andujar, Kusha Davar, Paul Holtom, Noah Wald-Dickler, Marco A. Lorio, Jose Gaviria, Vivian Chu, Cameron R. Wolfe, Mehri S. McKellar, Sumaya Farran, Roque A. Diaz Wong, Tjark Schliep, Raphael Shaw, Pablo Tebas, Aaron Richterman, Michelle Aurelius, Leah Peterson, Ron Trible, Tyler Rehman, Rabeeya Sabzwari, Edward Hines, Trevor Birkey, Jamie King, Ali Farabi, Elizabeth Jenny-Avital, Lauren Touleyrou, Avnish Sandhu, Gretchen Newman, Divya Bhamidipati, Divya Bhamidipati, Karen Vigil, Melissa Caro, Koury Banowski, Tanyanyiwa W. Chinyadza, Jaclyn Rosenzweig, Michelle S. Jones, Jose F. Camargo, Ketzela J. Marsh, Eugene W. Liu, Richelle Guerrero-Wooley, Paul Pottinger

**Affiliations:** ^1^CDC Monkeypox Emergency Response Team; ^2^Epidemic Intelligence Service, CDC; ^3^Minnesota Department of Health; ^4^Vermont Department of Health; ^5^Harris County Public Health, Houston, Texas; ^6^Texas Department of State Health Services; ^7^Baylor College of Medicine, Houston, Texas; ^8^Harris Health System, Bellaire, Texas; ^9^Emory University School of Medicine, Atlanta, Georgia; ^10^Infectious Diseases Specialists of Atlanta, Decatur, Georgia; ^11^Georgia Department of Public Health; ^12^Chicago Department of Public Health, Chicago, Illinois; ^13^Rush University System for Health, Chicago, Illinois, ^14^Los Angeles County Department of Public Health, Los Angeles, California.; CDC; CDC; CDC; CDC; CDC; CDC; CDC; CDC; CDC; CDC; CDC; CDC; CDC; CDC; CDC; CDC; CDC; CDC; CDC; CDC; CDC; CDC; CDC; Columbia University Irving Medical Center-New York Presbyterian Hospital, New York, New York; INOVA Fairfax Hospital, Falls Church, Virginia; INOVA Fairfax Hospital, Falls Church, Virginia; INOVA Fairfax Hospital, Falls Church, Virginia; INOVA Fairfax Hospital, Falls Church, Virginia; INOVA Fairfax Hospital, Falls Church, Virginia; Johns Hopkins Hospital, Baltimore, Maryland; Johns Hopkins Hospital, Baltimore, Maryland; Johns Hopkins Hospital, Baltimore, Maryland; Johns Hopkins Hospital, Baltimore, Maryland; Regional One Health-Memphis; Regional One Health-Memphis, Memphis, Tennessee; Westchester Medical Center, Valhalla; Westchester Medical Center, Valhalla; Bronxcare Health System, New York, New York; The Ohio State University, Columbus, Ohio; The Ohio State University, Columbus, Ohio; Mount Sinai School of Medicine, New York, New York; Mount Sinai School of Medicine, New York, New York; Mount Sinai School of Medicine, New York, New York; Osceola Regional Medical Center, Kissimmee, Florida; University of Chicago School of Medicine, Chicago, Illinois; NYC Health + Hospitals/Bellevue Hospital Center, New York, New York; NYC Health + Hospitals/Elmhurst Hospital Center, New York, New York; Cleveland Clinic, Cleveland, Ohio; University of Maryland Medical Center, Baltimore, Maryland; University of Maryland School of Medicine, Baltimore, Maryland; UT Southwestern Medical Center, Dallas, Texas; UT Southwestern Medical Center, Dallas, Texas; UT Southwestern Medical Center, Dallas, Texas; UT Southwestern Medical Center, Dallas, Texas; UT-Southwestern Medical Center and Parkland Hospital and Health System, Dallas, Texas; Penrose-St. Francis Hospital, Colorado Springs, Colorado; UC Health University of Colorado Hospital, Aurora, Colorado; Tufts Medical Center, Boston, Massachusetts; LAC+USC Medical Center, Los Angeles, California; LAC+USC Medical Center, Los Angeles, California; LAC+USC Medical Center, Los Angeles, California; Providence St. Patrick Hospital, Missoula, Montana; Baptist Hospital, Miami, Florida; Duke University School of Medicine, Durham, North Carolina; Duke University School of Medicine, Durham, North Carolina; Duke University School of Medicine, Durham, North Carolina; DFW Infectious Diseases, Lewisville, Texas; Methodist Hospital, San Antonio, Texas; NYC Health + Hospitals/Harlem Hospital Center, New York, New York; NYC Health + Hospitals/Harlem Hospital Center, New York, New York;; University of Pennsylvania, Philadelphia, Pennsylvania; University of Pennsylvania, Philadelphia, Pennsylvania; North Carolina Office of the Chief Medical Examiner; Salina Family Healthcare Center, Salina, Kansas; Northside Hospital, Atlanta, Georgia; VA Hospital, Chicago, Illinois; VA Hospital, Chicago, Illinois; VA Hospital, Chicago, Illinois; University of Wisconsin Medical Center, Milwaukee, Wisconsin; University Medical Center of Southern Nevada, Las Vegas, Nevada; University Medical Center of Southern Nevada, Las Vegas, Nevada; NYC Health + Hospitals/Jacobi Medical Center, New York, New York; Wayne State University, Detroit, Michigan; Wayne State University, Detroit, Michigan; Wayne Health, Detroit, Michigan; University of Texas Medical Center, Austin, Texas; McGovern Medical School at UTHealth, Houston, Texas; McGovern Medical School at UT Health, Houston, Texas; HCA Florida Mercy Hospital, Miami, Florida; Mercy Health, Toledo, Ohio; Mercy Health, Toledo, Ohio; Jefferson Health, Philadelphia, Pennsylvania; Piedmont Hospital, Atlanta, Georgia; Miller School of Medicine, Miami, Florida; University of Minnesota, Minneapolis and Saint Paul, Minnesota; Loma Linda University Health, Loma Linda, California; Loma Linda University Health, Loma Linda, California; University of Washington, Seattle, Washington

As of October 21, 2022, a total of 27,884 monkeypox cases (confirmed and probable) have been reported in the United States.^§^ Gay, bisexual, and other men who have sex with men have constituted a majority of cases, and persons with HIV infection and those from racial and ethnic minority groups have been disproportionately affected (*1*,*2*). During previous monkeypox outbreaks, severe manifestations of disease and poor outcomes have been reported among persons with HIV infection, particularly those with AIDS (*3*–*5*). This report summarizes findings from CDC clinical consultations provided for 57 patients aged ≥18 years who were hospitalized with severe manifestations of monkeypox^¶^ during August 10–October 10, 2022, and highlights three clinically representative cases. Overall, 47 (82%) patients had HIV infection, four (9%) of whom were receiving antiretroviral therapy (ART) before monkeypox diagnosis. Most patients were male (95%) and 68% were non-Hispanic Black (Black). Overall, 17 (30%) patients received intensive care unit (ICU)–level care, and 12 (21%) have died. As of this report, monkeypox was a cause of death or contributing factor in five of these deaths; six deaths remain under investigation to determine whether monkeypox was a causal or contributing factor; and in one death, monkeypox was not a cause or contributing factor.** Health care providers and public health professionals should be aware that severe morbidity and mortality associated with monkeypox have been observed during the current outbreak in the United States (*6*,*7*), particularly among highly immunocompromised persons. Providers should test all sexually active patients with suspected monkeypox for HIV at the time of monkeypox testing unless a patient is already known to have HIV infection. Providers should consider early commencement and extended duration of monkeypox-directed therapy^††^ in highly immunocompromised patients with suspected or laboratory-diagnosed monkeypox.^§§^ Engaging all persons with HIV in sustained care remains a critical public health priority.

During the ongoing monkeypox outbreak, CDC has provided consultation upon request to jurisdictions and clinicians treating patients with monkeypox.^¶¶^ This report describes the patients from these consultations who were aged ≥18 years and were hospitalized with probable or confirmed monkeypox during August 10–October 10, 2022; the report includes detailed histories for three patients who experienced severe manifestations of monkeypox. CDC obtained data on patient demographic characteristics, clinical course, and outcomes during consultation with health departments or providers. Patient permission for the use of clinical images was obtained. This activity was reviewed by CDC and was conducted consistent with applicable federal law and CDC policy.***

During August 10–October 10, 2022, CDC provided consultation for 57 patients aged ≥18 years who were hospitalized with severe manifestations of monkeypox (Table 1). Among 57 patients, 54 (95%) were male, and the median age was 34 years (range = 20–61 years). Forty-seven (82%) had HIV infection; among these patients, 31 (72%) of 43 with a known CD4 count had <50 CD4 cells/mm^3^ (Table 2). Two patients (4%), one of whom had HIV infection, were undergoing chemotherapy for a hematologic malignancy, three (5%) were solid organ transplant recipients, and three (5%) were pregnant. Overall, most patients were Black (68%), and 13 (23%) were experiencing homelessness.^†††^

**TABLE 1:** Characteristics of hospitalized patients with severe manifestations of monkeypox* for whom CDC provided clinical consultation (N = 57) — United States, August 10–October 10, 2022

Characteristic	No. (%)
**Median age, yrs (range)**	34 (20–61)
**Sex**	
Male	54 (94.7)
**Race and ethnicity**	
Black or African American, non-Hispanic	39 (68.4)
White, non-Hispanic	8 (14.0)
Hispanic or Latino	8 (14.0)
Asian, non-Hispanic	1 (1.8)
Multiple races, non-Hispanic	1 (1.8)
**Experiencing homelessness** **†**	13 (22.8)
**Any immunocompromising condition** **§**	51 (89.5)
HIV infection	47 (82.5)
History of solid organ transplantation	3 (5.3)
Hematologic malignancy (current chemotherapy)	2 (3.5)
**Pregnant**	3 (5.3)
**Clinical manifestation**¶	
Dermatologic	57 (100.0)
Mucosal**	39 (68.4)
Pulmonary	12 (21.1)
Ocular	12 (21.1)
Deep tissue (muscle or bone)	5 (8.8)
Neurologic	4 (7.0)
**Monkeypox-directed therapy**††	
Tecovirimat (oral)	53 (93.0)
Tecovirimat (intravenous)	37 (64.9)
VIGIV	29 (50.9)
Cidofovir††	13 (22.8)
**Received ICU-level care**	17 (29.8)
**STI coinfection** **§§**	16 (28.1)

**Abbreviations:** ICU = intensive care unit; STI = sexually transmitted infection, VIGIV = vaccinia immune globulin intravenous.

* Severe manifestations of monkeypox include, but are not limited to, the clinical findings listed at https://emergency.cdc.gov/han/2022/han00475.asp.

† Homelessness was defined by the clinician caring for the patient and included the experience of both sheltered and unsheltered homelessness. https://www.cdc.gov/ddid/homelessness/definition.html

§ One patient had HIV infection and was receiving chemotherapy for a hematologic malignancy.

¶ Patients could experience more than one clinical manifestation.

** Mucosal involvement might include oral, urethral, rectal, vaginal, or other lesions.

^††^ Patients could receive more than one treatment. All patients who received VIGIV or cidofovir also received tecovirimat.

§§ STI coinfection included concurrent diagnosis of syphilis, gonorrhea, chlamydia, herpes simplex virus type 2, or shigellosis.

**TABLE 2:** Laboratory and treatment characteristics of hospitalized patients with HIV infection and severe monkeypox* for whom CDC provided clinical consultation (N = 47) — United States, August 10–October 10, 2022

Characteristic (no. with information available)	No. (%)
**HIV CD4, cells/mm^3^ (43)**	
<50	31 (72.1)
50–200	9 (20.9)
>200	3 (7.0)
**HIV Treatment (47)**	
On ART at the time of monkeypox diagnosis	4 (8.5)

**Abbreviation**: ART = antiretroviral therapy.

* Severe manifestations of monkeypox include, but are not limited to, the clinical findings listed at https://emergency.cdc.gov/han/2022/han00475.asp.

All patients had severe dermatologic manifestations, and 39 (68%) also had severe mucosal lesions (Table 1). Some experienced involvement of other organs, including the lungs (12, 21%), eyes (12, 21%), and brain or spinal cord (four, 7%). Overall, 53 (93%) patients received oral tecovirimat, and 37 (65%) received intravenous tecovirimat; 29 (51%) patients received vaccinia immune globulin intravenous (VIGIV),^§§§^ and 13 (23%) received intravenous cidofovir. All patients who received cidofovir or VIGIV also received tecovirimat. Seventeen (30%) patients received ICU-level care and 12 (21%) died: monkeypox was a cause of death or contributing factor in five of these cases, six deaths remain under investigation to determine whether monkeypox was a causal or contributing factor, and in one death, monkeypox was not a cause or contributing factor.

## Representative Case Descriptions

**Patient A.** In August 2022, a Hispanic or Latino man in his 20s with no known past medical history was evaluated at an emergency department for back pain and a diffuse rash (location not specified). He was prescribed a course of prednisone for the back pain. Swabs were taken from the lesions to test for *Orthopoxvirus* (OPXV) by PCR, and the results were positive two days later. Over the next week, the patient’s rash progressed to involve his entire body. He was admitted to a hospital after being evaluated for dyspnea on exertion, dry cough, persistent back pain, and painful left neck swelling. On admission, he was febrile (102.8°F [39.3°C]), and he had a diffuse rash with central ulcerations as well as eschars on his face, trunk, and extremities; oral lesions; and a left neck mass. Laboratory results indicated a positive test result for HIV (CD4 = 79 cells/mm^3^, CD4 T-lymphocyte percentage 3%). According to state reporting, the patient had received a positive HIV test result in 2020 but was subsequently lost to follow-up. A computed tomography scan of his neck identified a 6.9 x 7.7 x 9.8–cm mass and extensive bilateral cervical lymphadenopathy. On hospital day 2, the patient became somnolent and was transferred to ICU; the next day, he was intubated for airway protection and received intravenous tecovirimat. He developed vasopressor-resistant hypotension, experienced a seizure, and went into kidney failure. During the next several days he was treated with vasopressors, antiepileptics, antibiotics, and antifungals, and required cardiopulmonary resuscitation. An extensive evaluation for infectious agents other than OPXV and HIV was negative. On the second day in ICU, he received 1 dose of VIGIV. Two days later, a brain scan indicated poor perfusion. The family elected to transition the patient to comfort measures. He was terminally extubated. An autopsy was conducted, with pathologic findings of necrosis in multiple tissues consistent with diffuse monkeypox. Immunohistochemistry testing demonstrated extensive orthopoxviral antigen in multiple tissues. Cytomegalovirus antigen was also detected in some tissues.^¶¶¶^

**Patient B.** In July 2022, a Black man in his 30s with AIDS (CD4 <10 cells/mm^3^) and not receiving ART developed a rash on his face, head, back, and genitals. At multiple subsequent clinic visits, he was tested and treated for gonorrhea, chlamydia, and syphilis; however, his genital lesions progressed, and he experienced phimosis and urinary retention for which he was admitted to a hospital 4 weeks after his rash began. A lesion swab taken the day of admission tested positive for *Monkeypox virus* (MPXV) DNA by PCR. The patient was discharged with a urinary catheter and 14 days of oral tecovirimat (Supplementary Figure 1; https://stacks.cdc.gov/view/cdc/121838). His skin lesions initially improved, but then spread, coalesced, and developed central necrosis (Figure) (Supplementary Figure 2, https://stacks.cdc.gov/view/cdc/121835). A suprapubic catheter was placed because of continued need for urinary catheterization. Approximately 10 days after discharge, the patient was readmitted with malaise, poor appetite, weight loss, and new hand and penile lesions. During a 15-day hospitalization, the patient was found to have methicillin-resistant *Staphylococcus aureus* bacteremia. He was transferred to ICU because of atrial fibrillation with rapid ventricular response. In ICU he was treated with intravenous tecovirimat, 2 doses of VIGIV, and antimicrobials. Conjunctivitis developed and was treated with trifluridine and antibacterial eye drops. The patient was discharged on oral tecovirimat and ART and with a suprapubic catheter. During week 7 of oral tecovirimat, he was readmitted because of progressive necrotic lesions with bacterial superinfection on the left hand, left eyelid lesions with periorbital swelling, and a right ear canal lesion associated with drainage and decreased hearing. He was restarted on intravenous tecovirimat and continues this treatment as of this report.

**FIGURE:**
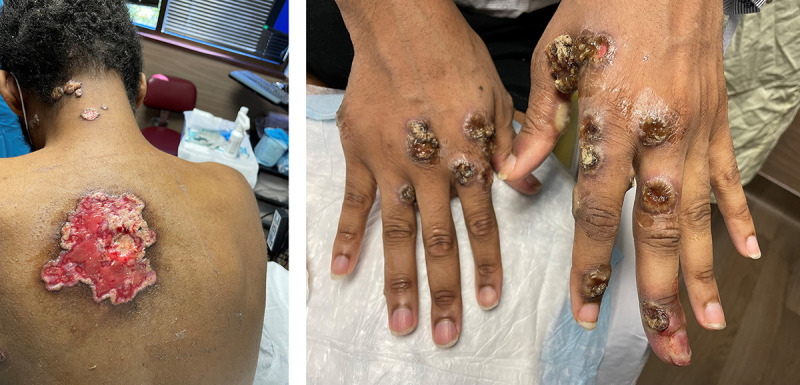
Disseminated lesions on the back and hands of a patient* with severe monkeypox — United States, August 10–October 10, 2022 Photos/Alexandra Dretler * Patient has consented to the publication of these photographs.

**Patient C.** In July 2022, a non-Hispanic White man in his 40s with AIDS (CD4 <10 cells/mm^3^) and not receiving ART was evaluated for a rash on his face, torso, hands, feet, and perianal area; lesion swabs tested positive for MPXV DNA by PCR. He was admitted to a hospital for pain control and received oral tecovirimat and ART. The patient experienced pain relief and was discharged after 7 days to complete 14 days of tecovirimat. However, his housing and food situations were unstable, and absorption of oral tecovirimat is dependent on concurrent intake of a full, fatty meal. Approximately 3 weeks after discharge, he was readmitted with coalescing, painful, and necrotic lesions on his hands and feet. Despite treatment with oral and intravenous tecovirimat for >4 weeks, 2 doses of cidofovir, 1 dose of VIGIV, and multiple antibiotics, progressive tissue necrosis led to debridement of the soft tissues of the right index finger and amputation of the right fourth toe. Gradually, the monkeypox lesions regressed. He was discharged but was readmitted 1 week later for unresolved lesions and severe pain. He received a second dose of VIGIV and remains hospitalized on oral tecovirimat and ART as of this report. 

## Discussion

Although most monkeypox cases during the ongoing outbreak have been self-limited (*2*,*8*), this report highlights the occurrence of severe manifestations of monkeypox in the United States, particularly in persons with AIDS. In this cohort of patients hospitalized with monkeypox and for whom clinicians or jurisdictions sought consultations with CDC, nearly one third (30%) received ICU-level care, and 21% of patients died, including several deaths that remain under investigation to determine the cause of death. Most patients eventually received tecovirimat, but some experienced delays of up to 4 weeks between initial care-seeking for monkeypox symptoms and initiation of monkeypox-directed therapy. For patients with suspected or laboratory-diagnosed monkeypox who are at risk for severe disease (particularly those with AIDS and other types of severe immunocompromise), health care providers should consider starting monkeypox-directed therapy early, potentially before receipt of monkeypox testing results or before severe manifestations are observed. In patients with severe disease, or with ongoing disease despite treatment, providers should consider extending tecovirimat treatment beyond 14 days and escalating therapy to include cidofovir or VIGIV if clinically indicated (*9*). For patients with HIV disease who are not on ART, clinicians should initiate ART as soon as possible, regardless of CD4 cell count.**** Health care providers should test all sexually active patients with suspected monkeypox for HIV at the time of testing for monkeypox unless a patient is already known to have HIV infection.

Most patients in this cohort were Black men, and nearly one quarter of cases occurred in persons experiencing homelessness. These findings likely reflect inequities in access to resources for the prevention, early diagnosis, and treatment of HIV infection, as well as missed opportunities to engage groups that have been socially or economically marginalized.^††††^ Public health outreach should strive to engage all persons with HIV infection in care and to increase access to monkeypox vaccination, diagnosis, and treatment. To accomplish these goals, it is critical to leverage existing HIV and sexually transmitted infection program resources and prioritize communities disproportionately affected by HIV (*1*). Collaboration with homeless services providers can help engage persons who are experiencing homelessness in prevention and treatment services for HIV and monkeypox.

The findings in this report are subject to at least four limitations. First, cases were passively identified by CDC through consultations requested by clinicians or jurisdictions and might not be representative of all patients with severe monkeypox. Second, this report only included outcomes occurring during the study period; therefore, deaths occurring after this period were not included. Third, observed morbidity and mortality might have been related to factors apart from or in addition to monkeypox, including HIV-related opportunistic infections. Finally, conclusions about the effectiveness of monkeypox treatments cannot be inferred from these observational data.

The occurrence of severe manifestations of monkeypox in patients who were most commonly immunocompromised because of AIDS highlights the importance of engaging all persons with HIV in sustained care and ending the HIV epidemic. Clinicians should consider close clinical monitoring, early treatment with available medical countermeasures, and extension or escalation of therapy as indicated in patients with or at risk for severe monkeypox. Ensuring equitable access to resources for the diagnosis, treatment, and prevention of HIV and monkeypox remains a vital public health priority.

**Summary**

**What is already known about this topic?**

Severe manifestations of monkeypox in immunocompromised persons have been observed in previous outbreaks.

**What is added by this report?**

During August–October 2022, CDC provided clinical consultation for 57 hospitalized patients with severe manifestations of monkeypox, most of whom were Black men with AIDS. Delays were observed in initiation of monkeypox-directed therapies. Twelve patients died, and monkeypox was a cause of death or contributing factor in five patients to date, with several other deaths still under investigation.

**What are the implications for public health practice?**

Clinicians should consider early treatment with available therapeutics for those at risk for severe monkeypox disease, particularly patients with AIDS. Engaging all persons with HIV in care remains a critical public health priority.

## Footnotes

* These authors contributed equally to this report.

§
https://www.cdc.gov/poxvirus/monkeypox/response/2022/us-map.html

¶ A list of severe manifestations of monkeypox can be found at https://emergency.cdc.gov/han/2022/han00475.asp.

** During the study period and as of October 21, 2022, CDC was notified by state and local jurisdictions of five decedents whose death certificates included monkeypox as a cause of death or contributing factor, six decedents whose cause of death is still under active investigation, and one decedent in whom the death was not monkeypox-related. Additional monkeypox cases involving severe disease or death might not be included in this report if CDC has not yet been notified about the case or if the case occurred outside of the study period. https://www.cdc.gov/poxvirus/monkeypox/response/2022/index.html

††
https://www.cdc.gov/poxvirus/monkeypox/clinicians/treatment.html

§§
https://www.cdc.gov/poxvirus/monkeypox/clinicians/case-definition.html

¶¶ CDC offers a monkeypox clinical consultation service for the ongoing monkeypox outbreak. Health care providers seeking additional clinical guidance can contact the CDC Emergency Operations by phone (770-488-7100) or by email (eocevent482@cdc.gov).

*** 45 C.F.R. part 46.102(l)(2), 21 C.F.R. part 56; 42 U.S.C. Sect. 241(d); 5 U.S.C. Sect. 552a; 44 U.S.C. Sect. 3501 et seq.

††† Homelessness was defined by the clinician caring for the patient and included the experience of both sheltered and unsheltered homelessness. https://www.cdc.gov/ddid/homelessness/definition.html

§§§ Tecovirimat, an FDA-approved treatment for smallpox, demonstrated efficacy against monkeypox in animal studies. Interim CDC guidance currently recommends that tecovirimat be considered in patients with severe monkeypox, those at high risk for severe disease, or those whose infection involves anatomic areas where monkeypox virus infection might constitute a special hazard (e.g., the eyes, pharynx, genitals, or anus). VIGIV has been used to treat complications from vaccinia vaccination. CDC holds an expanded access investigative new drug protocol that allows the use of VIGIV for the treatment of orthopoxviruses (including monkeypox) in an outbreak. https://www.fda.gov/media/78174/download

¶¶¶ Microscopic examination of autopsy tissues at the hospital and CDC showed findings consistent with diffuse monkeypox in specimens from a foot skin lesion, abdomen skin lesion, lip, vocal cord, lung, esophagus, mediastinal lymph nodes, and rectal mass. CDC performed immunohistochemistry testing that demonstrated extensive orthopoxviral antigen in multiple skin and mucosal tissues and in liver, pancreas, testis, adrenal gland, lung, and multiple lymph nodes. Cytomegalovirus antigen was also detected in a subset of skin, mucosal, and lymph node tissues.

****
https://www.cdc.gov/hiv/clinicians/treatment/treatment-clinicians.html

††††
https://www.hiv.gov/hiv-basics/overview/about-hiv-and-aids/who-is-at-risk-for-hiv

## References

[1] Curran KG, Eberly K, Russell OO, ; Monkeypox, HIV, and STI Team. HIV and sexually transmitted infections among persons with monkeypox—eight U.S. jurisdictions, May 17–July 22, 2022. MMWR Morb Mortal Wkly Rep 2022;71:1141–7. 10.15585/mmwr.mm7136a1 36074735 PMC9470220

[2] Philpott D, Hughes CM, Alroy KA, ; CDC Multinational Monkeypox Response Team. Epidemiologic and clinical characteristics of monkeypox cases—United States, May 17–July 22, 2022. MMWR Morb Mortal Wkly Rep 2022;71:1018–22. 10.15585/mmwr.mm7132e3 35951487 PMC9400536

[3] Ogoina D, Iroezindu M, James HI, Clinical course and outcome of human monkeypox in Nigeria. Clin Infect Dis 2020;71:e210–4. 10.1093/cid/ciaa143 32052029

[4] Yinka-Ogunleye A, Aruna O, Dalhat M, ; CDC Monkeypox Outbreak Team. Outbreak of human monkeypox in Nigeria in 2017–18: a clinical and epidemiological report. Lancet Infect Dis 2019;19:872–9. 10.1016/S1473-3099(19)30294-4 31285143 PMC9628943

[5] Selik RM, Mokotoff ED, Branson B, Revised surveillance case definition for HIV infection—United States, 2014. MMWR Morb Mortal Wkly Rep 2014;63(No. RR-3). 24717910

[6] Cash-Goldwasser S, Labuda SM, McCormick DW, ; CDC Monkeypox Clinical Escalations Team. Ocular monkeypox—United States, July–September 2022. MMWR Morb Mortal Wkly Rep 2022;71:1343–7. 10.15585/mmwr.mm7142e1 36264836 PMC9590292

[7] Pastula DM, Copeland MJ, Hannan MC, Two cases of monkeypox-associated encephalomyelitis—Colorado and the District of Columbia, July–August 2022. MMWR Morb Mortal Wkly Rep 2022;71:1212–5. 10.15585/mmwr.mm7138e1 36136957 PMC9531567

[8] Thornhill JP, Barkati S, Walmsley S, ; SHARE-net Clinical Group. Monkeypox virus infection in humans across 16 countries—April–June 2022. N Engl J Med 2022;387:679–91. 10.1056/NEJMoa2207323 35866746

[9] CDC. Monkeypox: Information for healthcare providers on obtaining and using TPOXX (tecovirimat) for treatment of monkeypox. Atlanta, GA: US Department of Health and Human Services, CDC; 2022. (Accessed October 17, 2022). https://www.cdc.gov/poxvirus/monkeypox/clinicians/obtaining-tecovirimat.html

